# Why Ubiquitin Has Not Evolved

**DOI:** 10.3390/ijms18091995

**Published:** 2017-09-16

**Authors:** Douglas C. Allan, James C. Phillips

**Affiliations:** 1 Corning Inc., Division Science &Technology, Corning, New York, NY 14831, USA; AllanDC@Corning.com; 2 Department of Physics and Astronomy, Rutgers University, Piscataway, NJ 08854, USA

**Keywords:** intracellular protein degradation, deubiquitinating enzymes, self-organization, allostery, network kinetics

## Abstract

Ubiquitin, discovered less than 50 years ago, tags thousands of diseased proteins for destruction. It is small (only 76 amino acids), and is found unchanged in mammals, birds, fish, and even worms, indicating that ubiquitin is perfect. Key features of its functionality are identified here using critical point thermodynamic scaling theory. These include synchronized pivots and hinges, a stabilizing central pivot, and Fano interference between first- and second-order elements of correlated long-range (allosteric) globular surface shape transitions. Comparison with its closest relative, 76 amino acid Nedd8, shows that the latter lacks all these features. A cracked elastic network model is proposed for the common target shared by many diseased proteins.

## 1. Introduction

Between the 1960s and 1980s, most life scientists focused their attention on protein creation by DNA and the translation of its coded information. Protein degradation was a neglected area, considered to be a nonspecific, dead-end process. Although it was known that proteins do turn over, the large extent and high specificity of the process, whereby distinct proteins have half-lives that range from a few minutes to several days, was not appreciated. The discovery of the complex cascade of the ubiquitin pathway revolutionized the field [1]. Today attention continues to be focused on DNA-based personalized medicine, but the very size of DNA makes this a challenging prospect. 

The extent and especially the depth of our understanding of individual proteins continue to grow in unexpected ways. Ubiquitin, discovered as the protein that targets other proteins for degradation [2], is the subject of multiple international conferences annually. Excellent extensive reviews of ubiquitination and targeting are available online, for example, in the Proteasome Wiki. Here we apply theoretical tools that show that ubiquitin has something very special in combination with hemoglobin: it confirms a remarkably prescient conjecture by Hopfield in 1973 [3] that identified a fundamental feature that makes both proteins necessary metabolic constituents of all tissues. He suggested that “a quantitative understanding of co-operativity in hemoglobin must include a description of where and how the free energy of co-operation is stored in the molecule. One extreme possibility is that the free energy is stored as small amounts of strain energy in many bonds, so that all bonds are almost normal”. His “linear distributed energy model” is consistent with Fano’s configuration interaction model of atomic spectra [4]. Specifically, it supports the existence of, and interference between, strain waves localized at hemes with continuum strain waves associated with tetrameric globin interfaces in hemoglobin, even over long times associated with O_2_ and CO ligand transport [5].

Here we will continue to use thermodynamic scaling and hydropathic waves [6] to analyze the functionality of ubiquitin. Whereas hemoglobin is a ~580 amino acid tetramer—a dimer of dimers of ~145 amino acid globins—ubiquitin is a small protein with only 76 amino acids. Given the weakly broken tetrahedral symmetry of hemoglobin, one could guess that the globins are coupled by hydropathic strain waves, so the linear [3] interference model is plausible, especially after it has been supported by parameter-free calculations [5,6]. Since ubiquitin is so small, why should we expect it to exhibit wave interference? The answer is that ubiquitin’s ability to identify all kinds of diseased proteins suggests that these protein networks share a common topological feature. Relative to stable healthy proteins, the strain fields of diseased proteins could be cracked, and interference between distributed continuum waves and waves localized at nanocracks [7] could be a second protein example of the Fano effect [4]. In crystalline science, such cracks are called dislocations. The presence of dislocations strongly influences many of the properties of materials

Since they are self-organized and viscoelastic, glass, granular, and protein networks share many thermodynamic properties [8,9,10,11,12]. Their structural phase transitions are generally mixtures of first-order and second-order transitions, and can have predominantly first-order or second-order character at different wave lengths. In glasses this distinction has been used to study dynamical relaxation [13]. In proteins the same distinction can be made by using two hydropathicity scales, for instance, the classical scale associated with first-order water-air unfolding enthalpy differences [14], and the modern second order scale associated with fractals derived from linearity of log-log plots of solvent associated surface areas as a function of segmental length [15]. Often the modern scale is more accurate, but exceptions may occur with enzyme interactions.

Another tool which has provided some striking results is the evolution of a protein, for instance, Hen Egg White [6]. However, ubiquitin is very special, because its 76 amino acid sequence has been found unchanged in mammals, birds, fish, and even invertebrate worms (search on Web-based BLAST, the Basic Linear Alignment Search Tool). Absent evolutionary guides, one still has the wave length as a probe for long-range (allosteric) interactions [13,16,17]. Protein interactions can be unusually strong at hydrophobic spots, so the length scale of globular surface roughness (in other words, the variance of hydropathic profiles) is a key tool for quantifying hydropathic waves and their interference.

All calculations are based on two hydropathic Ψ(amino acid) scales [14,15], linearly scaled to a common center and a common range [18] for each of the 20 amino acids. These two scales are derived in different ways, and describe different aspects of the internal interactions of amino acids in protein globules. The 1982 Kyte-Doolittle (KD) Ψ(amino acid) scale describes the short-range interactions of individual amino acids, such as would occur in protein unfolding. The 2007 Moret-Zebende Ψ(amino acid) scale describes long-range interactions, such as would occur in large proteins involving hundreds, or even thousands, of amino acids. The specific 76 amino acid sequence of ubiquitin is then converted to a triangular matrix Ψ(aa,*W*), where *W* is the length of a sliding window centered on each protein site. Variance is a standard statistical tool, available in EXCEL (Microsoft), where all calculations can be performed. We have studied the full range 1 ≤ *W* ≤ 75.

## 2. Results

Absence of evolution is the most striking feature of ubiquitin; thermodynamically, it is explained by saying that ubiquitin reached its critical point of functional perfection already even in worms. This conclusion can be tested by comparing the profiles of ubiquitin and Nedd8 (its closest sequence-alike (~70%) protein [1,2]). Nedd8 (also 76 amino acids) itself is interesting: it evolved very little from worm to mouse (BLAST sequence similarity high, ~92%), and remained unchanged in mammals from mouse to humans. The perfection of ubiquitin (76 amino acids) is reflected in the ~80 biological processes listed on its Uniprot page, compared to 9 for Nedd8, and 5 for hen egg white (148 amino acids, almost twice as large). 

**Figure 1:**
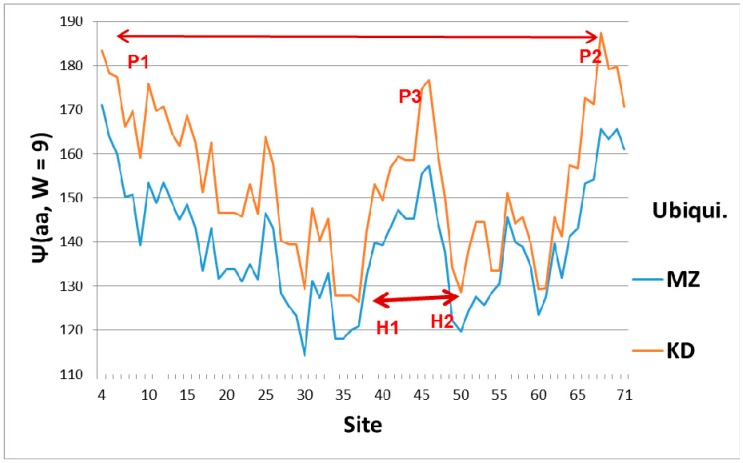
Hydropathic profiles with sliding window width *W* = 9 (smallest value associated with MZ scale, and highest resolution) for ubiquitin. Sites are numbered from N terminal to C terminal, and the two scale centers have been slightly offset for clarity, with larger values being more hydrophobic, and smaller values more hydrophilic. Hydroneutral is approximately 155 on both scales. The overall shape is roughly that of a symmetrical parabolic bowl, with a hydrophobic peak P3 ~45 functioning as an elastic pivot in the center. Thus the N and C terminal wings can swing separately, facilitating tagging the opposite sides of a crack in the strain field of a diseased protein. The average hydropathicity of ubiquitin is hydrophilic, which means it is elastically softer than average, which again facilitates tagging diseased cracks near the N and C terminals. The hydrophobic N (P1) and C (P2) terminal peaks are nearly level, which facilitates synchronous tagging, as are the hydrophilic hinges H1 and H2.

The broad features of the Ψ(aa,*W*) ubiquitin profiles with the first-order KD and second-order MZ scale are similar, as shown in Figure 1, which explains their orientation. The dominant feature of Ub is the two sets of level extrema, (P1,P2) and (H1,H2), which synchronize and accelerate tagging [6]. This figure also shows that a key role in ubiquitin is played by the central hydrophobic peak P3, which functions mechanically as an elastic pivot, separating two hydrophilic hinges. The significance of the hydrophobic peak is also clear in a hydrodynamic model, where peptide chain segment C_α_ center motion is described by vectors describing particle flow. A general theorem of vector fields decomposes them into solenoidal and irrotational components. The solenoidal components are connected to vortex centers, and here the hydrophobic peak is such a center. Its relative strength is larger using the first-order KD scale. Another way of explaining the dominance of the KD fluctuations is that the function of Ub is intrinsically first-order (the diseased protein is tagged once, and then destroyed by the ubiquitinase cascade [1]).

**Figure 2:**
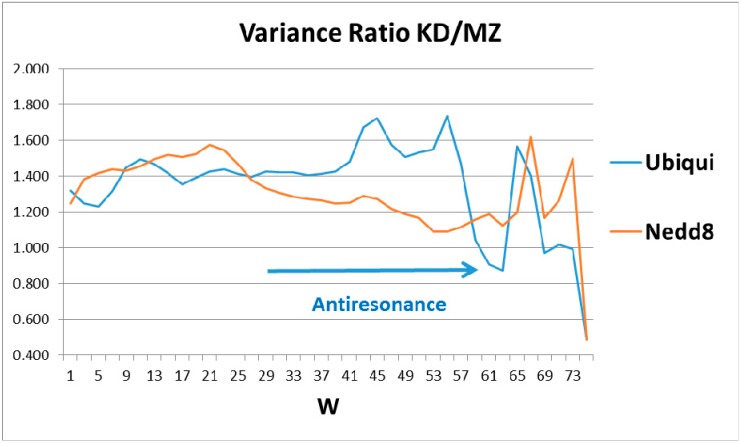
The human globular roughness, as a function of sliding window length, *W*, shows a rich structure for ubiquitin, but only weak structure for Nedd8. Here the structure above *W* 67 could be noise, but the two peaks centered on *W* = 45 and 55, and the sharp dip at 61–63, are genuine. Since ubiquitin is perfect, we expect that its second-order fine structure, monitored by the MZ variance, has become smooth (small variance), and that it is this MZ smoothness being small (or the KD roughness being large) that produces the two peaks in KD/MZ. Similarly, the 61–63 anti-resonance should be caused by a rough spot in MZ which evolution has not removed. This could be the two N- and C-wings of the overall profile (Figure 1) which are critical to its tagging opposing sides of target cracks.

The ratios of the globular roughnesses with the two scales are shown in Figure 2, whose caption discusses their structural details. Here one should note that globular surface roughness is a property currently fashionable in pure mathematics (differential geometry). It is related to volume properties by a generalized version of Stokes’ theorem, a standard part of advanced calculus. Spectroscopic data were the basis for Niels Bohr’s planetary model of the atom. It was not until 50 years later that it was realized that bilinear opto-electronic wave interference can be found in many carefully prepared liquid and solid samples [4], thus echoing Young’s 1803 two-slit diffraction experiment.

Most proteins’ functionality is complex and extremely nonlinear, so one might not expect to find bilinear wave interference in their properties, especially in hydropathically-shaped globular structures. Hemoglobin and ubiquitin are exceptions, the first identified (to our knowledge). The long-standing mystery of the molecular origin of collective heme α-β interactions is explained by interference of heme and interface-based hydropathic waves [5]. Here the perfection and functional generality of tagging strain field cracks of diseased proteins has led to the rich structure of ubiquitin in Figure 2, which is basically absent in Nedd8.

**Figure 3:**
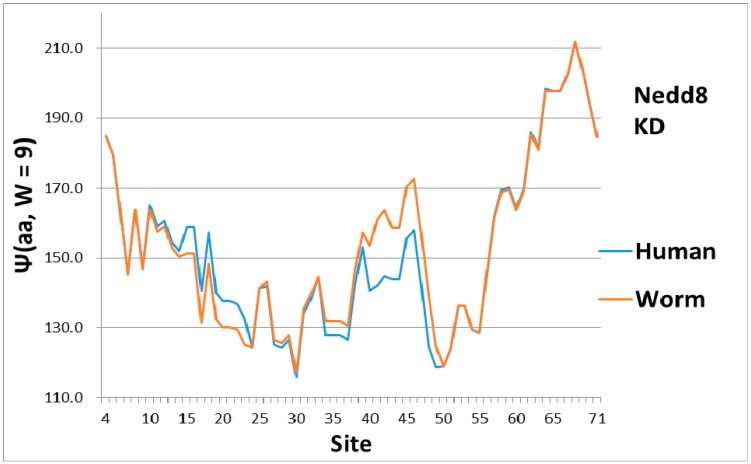
In the evolution of Nedd8 from worm to human, most of the changes appear to be first order (KD scale) and are concentrated in softening the central pivot. This contrasts strongly with distributed smoothing of ubiquitin in Figure 1. Note also that the C terminal here is much more hydrophobic than the N terminal, whereas the two ends are nearly equally hydrophobic (level) in Ub (Figure 1).

**Figure 4:**
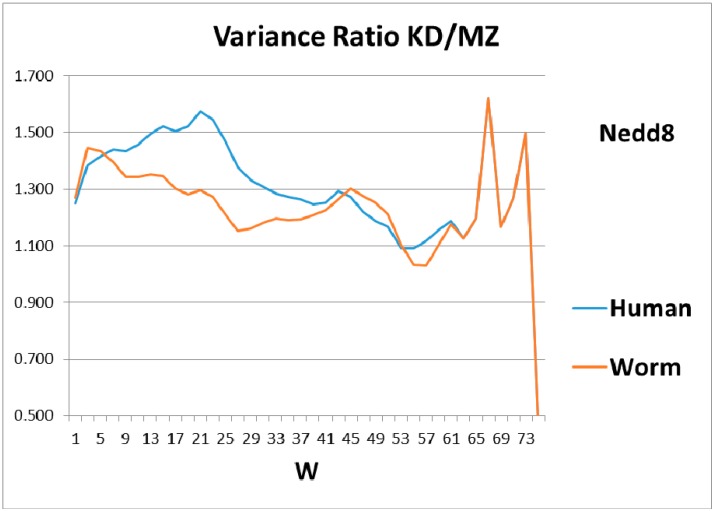
Comparison of the roughness (variance) of the hydropathic profiles for different sliding window wave lengths, *W*, for worm and human Nedd8 shows that evolution has mainly improved the shorter lengths, *W* ~20, while the peaks and anti-resonance seen in Figure 2 for ubiquitin are undeveloped.

As a check, there must be some indication of how the magical structure of ubiquitin has evolved, hidden in Nedd8 properties. There is, because although ubiquitin is perfect and has not evolved, its look-alike Nedd8 has. It turns out that the largest worm-human evolutionary changes in the Ψ(aa,*W* = 9) Nedd8 profiles occur in the first-order KD profile, which is shown in Figure 3. The variance profiles of human and worm Nedd8 are compared in Figure 4 and discussed there. The main point is that the nearly equal N and C terminal pivot hydrophobicities in Ub is absent in Nedd8.

### 2.1. Crack Model

With respect to the universality of ubiquitin tagging, cracks in a protein network can cause protein dysfunction, while preserving the protein fold. One can suppose that the cracks involve amino acid packing misfits, which would enhance a few irreversible first-order (strong) interactions, compared to many more reversible second-order (weak) allosteric interactions. Cracks can occur between rigid homologous protein domains, and may involve shear [19,20,21]. At such cracks there is relaxation at crack edges, stabilizing these edges. Thermal vibrational amplitudes near relaxed edges should be small, or alternatively there could be less thermal noise, facilitating long-range (large *W*) allosteric binding of ubiquitin along a crack. The crack length can be estimated to be about half of a typical domain length in very large hub enzymes, such as tyrosine kinases [22]. The latter domain lengths *L* are around 100 amino acids; halves of these lengths fit the resonances in Figure 2. The lengths *W* of the dominant localized modes associated with the cracks would also tend to be around *L*/2 for the softest modes. These same modes would interfere with conformational motions associated with protein functionality. Cracking leads to a counterintuitive catalytic effect of added denaturant on allosteric enzyme function in adenylate kinase [19,20]. More generally, cracks are an example of allosteric interactions which can be generated by removing only ~1% of the bonds [23].

If we assume that Ub binds to cracks, is there a local feature of Ub which would assist in crack binding? Presumably the crack represents a local reduction of amino acid density, and such a local crack would be filled with water. Looking at Figure 1, we see a deep hydrophilic minimum in the Ψ(aa,*W* = 9) Ub profile near sites 30–35 for both the first-order KD scale and the second-order MZ scale, with a similar minimum near 50–60. The combination of the two deep nearly-level minima could stabilize Ub tagging, and it is an appealing way of explaining Ub efficiency and universality.

### 2.2. Fano Interference

A word here is appropriate for physical scientists who are familiar with the Fano effect involving optical or elastic waves in physical nanostructures, but not proteins. Formally the Fano effect involves wave function amplitudes [4,7]. However, the essence of the Fano effect is duality: the localization of the strain wave on a small scale, which interferes with continuum states extended over a much larger scale. This duality is present in Ub binding to cracks, which should occur locally near the two hydrophilic N and C hinges (KD scale), compared to the extensive states which span the spacing between these binding points (MZ scale). Although biologists have not previously identified Fano effects, they are well aware of the importance of strain fields in mediating allosteric interactions [22]. Their interference in hemoglobin and ubiquitin is new.

Wave interference effects were first observed optically in the two-slit diffraction experiment in 1803 [24], and in standing surface waves of a viscous liquid in 1830 [25]. The notion that wave interference effects can be observed in connection with strain fields as well as in opto-liquid fields has become apparent from rapid advances in nanoscience [7]. Even so, the observation of such delicate effects in proteins in connection with hydropathic waves requires choosing just the right proteins as examples, to be combined with just the right bioinformatic scaling tools [15]. Although such ideal examples of wave interference effects are few, they form an exacting benchmark against which many other concepts and theoretical tools can be tested. The present approach to ubiquitylation, which emphasizes dynamic long wave length attractive interactions mediated by hydropathic waves, should be contrasted with contact (repulsive interaction) models based on static structures [26,27]. Perhaps the combination of the two approaches will yield new insights.

The emphasis in this article has been on manifestation of hydropathic wave interference through the Fano effect, which occurs on a large scale (*W* = 45, 55, 61) (see Figure 2). There are also indications of hydropathic elastic effects in NMR structural studies. The N (Arg 42–Val 70) CO and N (Arg 72–Gln 40) CO hydrogen bonds have maximally perturbed the interaction of ubiquitin with the signal transducing adaptor molecule-1 ubiquitin interacting motif (UIM) [26]. Looking at Figure 1, we see that these two bonds couple the two largest hydrophobic peaks. Hydrophilic extrema are also identified in this experiment: “a conformational change in ubiquitin is facilitated by changes in hydrogen bond lengths all over the protein backbone”. This behavior is characteristic of self-organized criticality [28]. One example cited, N (Ser 57–Pro 19) CO, corresponds to two local minima in Ψ(aa,*W*) for *W* = 21 on both the MZ and KD scales. This is a gratifying large-scale result, because *W* = 21 has been identified for strongly interacting membrane proteins as a best wavelength [29]. Note also that the formation of poly Ub chains [26,30,31] is analogous to critical opalescence [6]. It occurs so readily because ubiquitin is perfect.

Level sets of hydrophobic and hydrophilic extrema are seen in Ub (Figure 1), but not in Nedd8 (Figure 3). This is not accidental, because much larger level sets occur in the much larger (>1000 amino acids) protein, ubiquitin-activating enzyme Uba1 (E1) [32]. Generally, protein kinetics are faster when the hydropathic extrema form level sets [6]. It is most economical to destroy diseased proteins as fast as possible, in order to recycle their amino acids quickly. Overall, the evolutionary stability of Ub has made it possible to confirm the key role played by level sets in promoting accelerated kinetics.

## 3. Methods

Scales are a standard part of science, and scaling is a general method for comparing results obtained for different materials by different methods. Thermodynamic scaling is a new method invented for connecting protein amino acid sequences directly to protein functions, especially as the latter have evolved towards optimal functionality (reviewed in detail in [6]). There have been extensive attempts (MDS) to connect protein sequences to folded protein structures by using Newton’s equations of motion, suitably fitted to the very large library of static protein structures. While progress has been made on the “folding” problem, the second step, connecting structure to function, has remained largely unsolved, because of the limitations inherent to applying Newtonian methods (suitable to two bodies) to the many-body kinetic problem (thousands of atoms in proteins). Like MDS, thermodynamic scaling usually contains no adjustable parameters (the one parameter W* can usually be fixed by evolutionary comparisons), and the fractal compression of structural data enables it to treat a much wider range of problems with high accuracy. 

Many of the problems encountered by application of Newtonian methods to proteins are also encountered in the smaller problem of composition dependence of network glass properties. Solutions to this smaller problem are now well advanced [33], using post-Newtonian network methods whose development spanned several decades. The similarities between viscous glass melts and viscous protein conformational functions have also been known since the 1950’s [6], but a practical way of exploiting them involves temperature-dependent bonding constraints (glasses) or thermodynamic scales (proteins). The protein scale must be very accurate, and such accuracy has been achieved only recently [15]. Informally variance is known as “mean of square minus square of mean”, or for any function Φ

Var(Φ(aa,*W*)) = Σ((Φ(aa,*W*) − <Φ(aa,*W*)>)^2^ = ΣΦ(aa,*W*)^2^ – n(<Φ(aa,*W*)>)^2−^ (1)

where the sum is taken over n consecutive amino acid sites. In the context of the MZ Ψ scale [15], the variance measures the hydropathic roughness of the globular surface of the n sites of the protein chain segment of length *W*. The local or global roughness of different modular elements can affect dynamical functions, which should occur neither too fast nor too slowly, in order to synchronize with other protein motions [6].

## References

[1] Glickman M.H., Ciechanover A. (2002). The ubiquitin-proteasome proteolytic pathway: Destruction for the sake of construction. Physiol. Rev..

[2] Wilkinson K.D. (2005). The discovery of ubiquitin-dependent proteolysis. Proc. Nat. Acad. Sci. USA.

[3] Hopfield J.J. (1973). Relation between structure cooperativity and spectra in a model of hemoglobin action. J. Mol. Biol..

[4] Fano U. (1961). Effects of configuration interaction on intensities and phase shifts. Phys. Rev..

[5] Sachdeva V., Phillips J.C. (2016). Hemoglobin Strain Field Waves and Allosteric Functionality. https://arxiv.org/abs/1606.00795.

[6] Phillips J.C. (2016). Quantitative Molecular Scaling Theory of Protein Amino Acid Sequences, Structure, and Functionality. https://arxiv.org/abs/1610.04116.

[7] Miroshnichenko A.E., Flach S., Kivshar Y.S. (2010). Fano resonances in nanoscale structures. Rev. Mod. Phys..

[8] Boolchand P., Lucovsky G., Phillips J.C., Thorpe M.F. (2005). Self-organization and the physics of glassy networks. Phil. Mag..

[9] Ellenbroek W.G., Hagh V.F., Kumar A., Thorpe M.F., .van Hecke M. (2015). Rigidity loss in disordered systems: Three scenarios. Phys. Rev. Lett..

[10] Maxwell J.C. (1864). On the calculation of the equilibrium and stiffness of frames. Philos. Mag..

[11] Kauzmann W. (1948). The nature of the glassy state and the behavior of liquids at low temperatures. Chem. Rev..

[12] Gibbs J.H., DiMarzio E.A. (1958). Nature of the glassy transition and the glassy state. J. Chem. Phys..

[13] Angell C.A. (1991). Relaxation in liquids, polymers and plastic crystals–Strong fragile patterns and problems. J. Non Cryst. Solids.

[14] Kyte J., Doolittle R.F. (1982). A simple method for displaying the hydropathic character of a protein. J. Mol. Biol..

[15] Moret M.A., Zebende G.F. (2007). Amino acid hydrophobicity and accessible surface area. Phys. Rev..

[16] Berthier L., Biroli G., Bouchaud J.-P., Cipelletti L., El Masri D., L'Hôte D., Ladieu F., Pierno M. (2005). Direct experimental evidence of a growing length scale accompanying the glass transition. Science.

[17] Zhang Y., Glor E.C., Li M., Liu T., Wahid K., Zhang W., Robert A R., Zahra F. (2016). Long-range correlated dynamics in ultra-thin molecular glass films. J. Chem. Phys..

[18] Phillips J.C. (2009). Scaling and self-organized criticality in proteins: Lysozyme c. Phys. Rev..

[19] Phillips J.C. (2017). Giant hub Src and Syk tyrosine kinase thermodynamic profiles recapitulate evolution. Phys. A Stat. Mech. Appl..

[20] Miyashita O., Onuchic J.N., Wolynes P.G. (2003). Nonlinear elasticity, proteinquakes, and the energy landscapes of functional transitions in proteins. Proc. Nat. Acad. Sci. USA.

[21] Whitford P.C., Onuchic J.N., Wolynes P.G. (2008). Energy landscape along an enzymatic reaction trajectory: Hinges or cracks?. HFSP J..

[22] Mitchell M.R., Tlusty T., Leibler S. (2016). Strain analysis of protein structures and low dimensionality of mechanical allosteric couplings. Proc. Nat. Acad. Sci. USA.

[23] Rocks J.W., Pashine N., Bischofberge I., Goodrich C.P., Liu A.J., Nagel A.R. (2017). Designing allostery-inspired response in mechanical networks. Proc. Nat. Acad. Sci. USA.

[24] Young T. (1807). A Course of Lectures on Natural Philosophy and the Mechanical Arts: In Two Volumes.

[25] Faraday M. (1831). On a peculiar class of acoustical figures; and on certain forms assumed by groups of particles upon vibrating elastic surfaces. Philos. Trans. R. Soc. Lond..

[26] Komander D., Rape M. (2012). The ubiquitin code. Annu. Rev. Biochem..

[27] Sundd M. (2012). Conformational and dynamic changes at the interface contribute to ligand binding by ubiquitin. Biochemisty.

[28] Tang Q.Y., Zhang Y.Y., Wang J., Wang W., Chialvo D.R. (2017). Critical fluctuations in proteins native states. Phys. Rev. Lett..

[29] Phillips J.C. (2016). Catalytic Nucleation of Amyloid Beta and Hen Egg White Fibrils, and p53 Oligomerization. https://arxiv.org/abs/1606.00636.

[30] Preston G.M., Brodsky J. (2017). The evolving role of ubiquitin modification in endoplasmic reticulum-associated degradation. Biochem. J..

[31] Yau R., Rape M. (2016). The increasing complexity of the ubiquitin code. Nat. Cell Biol..

[32] Allan D.C., Phillips J.C. (2017). Evolution of the ubiquitin-activating enzyme Uba1 (E1). Phys. A Stat. Mech. Appl..

[33] Smedskjaer M.M., Hermansen C., Youngman R.E. (2017). Topological engineering of glasses using temperature-dependent constraints. MRS Bull..

